# Mutational consequences of perturbing DNA repair and chromatin state in *Arabidopsis*

**DOI:** 10.64898/2026.08.04.742834

**Published:** 2026-08-06

**Authors:** C. A. Meyer, R. J Schmitz

## Abstract

Mutation rate varies throughout an organism’s genome and correlates with many factors, including primary sequence, DNA methylation, gene content, chromatin accessibility, replication timing, and more. Prior work has shown that DNA repair pathways contribute to this variation by repairing certain regions more efficiently than others, sometimes through interactions with gene-associated histone modifications. However, little is known about the relative importance of different DNA repair pathways in shaping intragenomic variability in mutation rate, nor the mutational consequences of perturbing chromatin state. Here, we quantify somatic mutation rate in several DNA repair and chromatin-related *Arabidopsis* mutants using nanorate sequencing. We find that NER and MMR prevent a smaller fraction of mutations in transposable elements (TEs) compared to other regions of the genome, indicating these pathways are less efficient in heterochromatin. MMR appears to be more efficient in accessible chromatin regions, as its loss nearly abolishes the reduced mutation rate there. TC-NER is the only pathway with greater efficiency in genes than in non-genic non-TE regions, suggesting only TC-NER specifically targets genes. We assay six mutants for histone modifications/variants, but only one ( *h2a.w.7* ) displays an altered mutation rate. Instead, mutation rate is elevated in the chromatin remodeler mutant *ddm1* and two RNA-directed DNA methylation (RdDM) mutants. The RdDM mutants have a doubled overall mutation rate, but this increase is not localized to RdDM target regions, implicating a transcriptional change, genomic instability, or a secondary function in DNA repair.

## Full Text Availability

The license terms selected by the author(s) for this preprint version do not permit archiving in PMC. The full text is available from the preprint server.

